# Can we ask them? Self-reported care experiences in long-term facilities for Severe Mental Illness and Korsakoff’s syndrome

**DOI:** 10.1192/j.eurpsy.2026.10968

**Published:** 2026-07-31

**Authors:** E. Oudman, I. Gijsberts, K. Naburgh-Visser

**Affiliations:** 1 Parnassia, The Hague; 2 Experimental Psychology, Utrecht University, Utrecht; 3 Korsakoff Center of Expertise Slingedael; 4 Erasmus School of Health Policy & Management, Rotterdam; 5 Het Parkhuis, Salios, Dordrecht, Netherlands

## Abstract

**Introduction:**

Patients with Severe Mental Illness (SMI) and Korsakoff’s syndrome (KS) in long-term care facilities face severe cognitive, psychiatric, and somatic challenges. Traditionally, quality of care in these groups is assessed through proxies, as self-report is often considered unreliable. This risks excluding patient perspectives from evaluation and improvement of care.

**Objectives:**

The present study sought to evaluate whether patients with SMI and KS are able to reliably self-report their satisfaction with long-term care. A secondary aim was to compare patterns of satisfaction across these groups in order to identify both differences and common ground in their perspectives on care.

**Methods:**

A cross-sectional design was employed. Data were collected from 86 geriatric SMI patients and 167 KS patients across six specialized long-term care facilities in the Netherlands. All participants completed a validated 14-item questionnaire assessing satisfaction with care across three domains: *Autonomy* (e.g., privacy, freedom, personal choice), *Influence on Care* (e.g., being listened to, participation in decisions), and *Activity Level* (e.g., engagement in meaningful daily activities). Items were rated on a 10 cm visual analogue scale. Independent sample t-tests were performed to compare mean domain scores between groups, with p < .05 considered statistically significant.

**Results:**

Both SMI and KS patients were able to complete the questionnaire and provide consistent self-ratings, thereby challenging the assumption that self-report is not feasible in these populations. Overall satisfaction levels were moderate to high in both groups. Significant differences emerged between groups: SMI patients reported higher satisfaction with *Influence on Care*, suggesting a stronger sense of involvement in treatment planning and shared decision-making. In contrast, KS patients reported higher satisfaction with *Activity Level*, reflecting their engagement in structured routines and daily programs. Despite these differences, both groups emphasized the importance of *Autonomy* as a central component of care quality. This overlap highlights autonomy as a universal theme across EPA populations, even in the presence of severe cognitive and psychiatric impairments.

**Conclusions:**

Even highly vulnerable EPA populations can provide valid self-reports. While group differences reflect distinct care needs, the shared emphasis on autonomy underscores the importance of integrating patient-reported outcomes into routine evaluation and quality improvement in long-term psychiatric care.

**Disclosure of Interest:**

None Declared

